# Gut Microbiome Alterations in Patients With Thyroid Nodules

**DOI:** 10.3389/fcimb.2021.643968

**Published:** 2021-03-12

**Authors:** Ang Li, Tiantian Li, Xinxin Gao, Hang Yan, Jingfeng Chen, Meng Huang, Lin Wang, Detao Yin, Hongqiang Li, Runsheng Ma, Qiang Zeng, Suying Ding

**Affiliations:** ^1^ Health Management Center, The First Affiliated Hospital of Zhengzhou University, Zhengzhou, China; ^2^ Department of Thyroidology, The First Affiliated Hospital of Zhengzhou University, Zhengzhou, China; ^3^ Health Management Institute，The Second Medical Center & National Clinical Research Center for Geriatric Diseases, Chinese PLA General Hospital, Beijing, China

**Keywords:** thyroid nodule, thyroid function, gut microbiota, metagenomics, comparative genomics, gut-thyroid link

## Abstract

Thyroid nodules are found in nearly half of the adult population. Accumulating evidence suggests that the gut microbiota plays an important role in thyroid metabolism, yet the association between gut microbiota capacity, thyroid nodules, and thyroid function has not been studied comprehensively. We performed a gut microbiome genome-wide association study in 196 patients with thyroid nodules and 283 controls by using whole-genome shotgun sequencing. We found that participants with high-grade thyroid nodules have decreased number of gut microbial species and gene families compared with those with lower grade nodules and controls. There are also significant alterations in the overall microbial composition in participants with high-grade thyroid nodules. The gut microbiome in participants with high-grade thyroid nodules is characterized by greater amino acid degradation and lower butyrate production. The relative abundances of multiple butyrate producing microbes are reduced in patients with high-grade thyroid nodules and the relative abundances of L-histidine metabolism pathways are associated with thyrotropin-releasing hormone. Our study describes the gut microbiome characteristics in thyroid nodules and a gut-thyroid link and highlight specific gut microbiota as a potential therapeutic target to regulate thyroid metabolism.

## Introduction

The incidence of thyroid nodules (TN) is increasing worldwide, such that they are now present in 20–76% of the adult population (Hoang et al., 2007). Risk factors for the development of TN include age, sex, metabolic syndrome, and iodine intake (Dauksiene et al., 2017; Yao et al., 2018), and several human genome loci, including TRPM3, EPB41L3, and AP005059, are associated with TN (Hwangbo et al., 2018). The importance of thyroid function for appropriate metabolic regulation has been recognized for decades, is affected by numerous environmental factors (Sarne, 2000), and is partially genetically determined in humans (Taylor et al., 2015). However, the etiology of TN and the role of genetics in thyroid function remain to be fully elucidated.

Evidence is accumulating that the gut microbiota plays an important role in thyroid disorders such as Hashimoto’s thyroiditis (Fröhlich and Wahl, 2019), thyroid carcinoma (Zhao et al., 2018), Graves’ disease (Feng et al., 2018), and primary hypothyroidism (Su et al., 2020). Thyroid homeostasis may be regulated by the effects of the gut microbiota on the immune system (Knezevic et al., 2020) and the metabolism of micronutrients such as minerals (Docimo et al., 2020; Samimi and Haghpanah, 2020). The importance of the gut microbiome on human health has been evident for more than a decade, however, our understanding of its role in thyroid functions and common conditions such as thyroid nodules are still very limited. A recent study showed gut microbial dysbiosis in 18 patients with TN and an association between the microbiome and thyroid function (Zhang et al., 2019). However, the significance of these findings was restricted by the small number of patients studied and the insufficient resolution based on 16S ribosomal RNA (rRNA) gene amplicon sequencing (McLaren et al., 2019).

We enrolled 1,389 participants who underwent a thyroid ultrasound during a routine physical examination check in this study. After exclusions, 196 participants with thyroid nodules and 283 body mass index (BMI)- and age-matched controls were selected as a primary analysis cohort and whole genome shotgun sequencing was performed on their stool samples. On average, 12.5 Gb (± 3.3 Gb) high-quality non-human data were generated per sample. In total, we obtained 6.25 TB data for downstream analysis. Of the participants, 235 underwent serum thyroid function testing, including the measurement of free triiodothyronine (FT3), free thyroxine (FT4), and thyroid stimulating hormone (TSH) concentrations. Using these data, we aimed to define the gut microbial characteristics associated with TN and thyroid function and verify these associations by using *in vivo* and *in vitro* experiments.

## Materials and Methods

This study was approved by the ethics committee from the First Affiliated Hospital of Zhengzhou University (2018-KY-56 and 2018-KY-90). Informed consent was obtained from all the subjects.

### Study Population

Exclusion criteria were as follows: 1) Diagnosed diabetes, hyperthyroidism, hypothyroidism, or cancer. 2) BMI under 18 or over 30. 3) Use of the following medications: antibiotics (in the last 3 months), antivirals, thyroxine agent, proton pump inhibitors (PPI), anti-glycemic, anti-hypertensive, or anti-hyperlipidemic therapies. 4) Thyroid surgery, thyroid enlargement, lymphadenectasis, or any thyroid cancers. 5) Lacking TI-RADS assessment. 9) Abnormal thyroid functions outside of the normal ranges for FT3, FT4, and TSH of 3.28–6.47 pmol/L, 7.9–18.4 pmol/L, and 0.34–5.6 mU/L, respectively. The participants abstained from alcohol 3 days before the physical examination and fasted from 8:00 pm, and were water-free after midnight, the previous evening. Plavix and anticoagulant drugs including warfarin and aspirin were stopped a week before their examination.

### Sample Collection

Stool samples were obtained from the recruited participants. The samples were immediately stored at −20°C and frozen at −80°C within 30 min of collection before being transported to the laboratory. Fasting blood and stool samples were all obtained on the same day at the hospital before 10 am.

### Laboratory Tests

Lavender-top tubes containing EDTA as an anticoagulant were routinely used to collect 1–2 ml of venous blood after which the tubes were shaken up and down evenly five to eight times to prevent the blood from clotting. Yellow-top tubes containing separating gel and coagulant to accelerate coagulation were used to collect 2 ml of venous blood for the assessment of blood lipids, blood glucose, and liver function. Blood samples were analyzed using a Roche cobas 8000 automatic biochemical analyzer. Red-top tubes without anticoagulant were used to collect 2 ml of venous blood for thyroid function assays, including free triiodothyronine (FT3), free thyroxine (FT4), and thyroid stimulating hormone (TSH). After the blood draws, all the tubes were placed in specimen boxes, the data were entered into the hospital specimen transmission system, and the specimens were sent to the central clinical laboratory *via* a logistics trolley within 20 min. The standard of the normal range was our hospital’s built-in cutoff. An UniCel DxI 800 Immunoassay System from Beckman Coulter was used to assay the blood samples.

### Thyroid Scans

A Philips Affiniti 50 color Doppler ultrasound system was used to scan the thyroid gland with a linear array probe and a frequency of 5-12MHz. Participants took a conventional supine position with their heads back and down, and necks high to allow full exposure of the anterior neck area. Continuous top-down cross-section, longitudinal section, and multi-section scans were performed to record the overall thyroid and intrathyroidal nodules in detail. The description of the intrathyroidal nodules was verified by at least two ultrasonography clinicians, and covered characteristics including the site, echo, number, size, aspect ratio, margin, border, morphology, stun, internal structure, calcification, and blood flow signal of the nodules. The thyroid nodules were classified according to the Kwak-TIRADS criteria (Kwak et al., 2011).

### DNA Extraction, Shotgun Metagenomic Sequencing, and Quantity Control of Reads

DNA from a total of 1,034 fecal samples was extracted using the MagPure Stool DNA KF kit according to the manufacturer’s instructions. DNA nanoball (DNB) based DNA library construction and combinatorial probe-anchor synthesis (cPAS)-based shotgun metagenomic sequencing with 100 bp paired-end reads were applied to all samples (MGI2000, MGI, Shenzhen, China). Quality control (QC) of raw sequencing reads was applied to filter out low-quality reads using an overall accuracy (OA ≥ 0.8) control strategy as previously described (Fang et al., 2018). High-quality reads were aligned to hg19 using SOAPaligner/soap2 to filter out human reads (identity ≥ 0.9).

### Microbiome Composition Profiling

Taxonomic annotation and quantification was performed based on MetaPhlAn2 with default settings (Truong et al., 2015), generating gut microbial profiling that included bacteria, archaea, eukaryotes, and viruses. Taxon-specific community functional profiles were further generated using HUMAnN2 (the HMP Unified Metabolic Analysis Network 2) (Franzosa et al., 2018).

### Association Between Gut Microbiome and Thyroid Nodules or Thyroid Function

MaAsLin2 (Multivariate Association with Linear Models) (Mallick et al.) was used to evaluate the association between gut microbial species or function composition and thyroid nodules or thyroid functions using default parameters except for “-p 0.2,” while controlling for demographic covariates (gender, age, BMI). Multiple comparisons were controlled for using the BH method. Positive coefficients of microbial species or pathways with HTN group (or TSH, FT4, FT4) were considered to indicate positive associations.

### Assessment of Inner-Microbiome Interaction

We used Spearman’s correlation coefficient and microbiome relative abundance to identify inter-microbial interactions, i.e., interactions between pathways and species. We randomly subsampled 80% of all samples to bypass possible outliners and repeated this process 100 times, then used the mean Spearman’s correlation coefficient and mean p-value as the inner-interaction measurement.

### Statistical Analysis

Statistical analyses were performed using the R program, version 3.6.1. The demographic, laboratory test results, and lifestyle characteristics of the participants were summarized using a standardized statistical significance test method, where the test pathway mainly adopts one-way ANOVA and chi-square test (**Table S1**). Categorical variables were shown as counts and percentages [n (%), missing n (%)], and associations were tested using a chi-square test. Continuous variables were shown as mean (± standard deviation), and differences between groups were analyzed in detail using a one-way ANOVA and normality test (Shapiro.test), then a homogeneity test (Bartlett.test or Levene.test), and finally a parameter test (aov.test or kruskal.test) and a non-parametric test (oneway.test). A p-value of ≥0.05 was considered in accordance with normality and homogeneity, while a p-value of <0.05 was considered statistically significant. The R program “glmnet” package and analysis of variance model (AOV) was used to assess the correlation between microbial diversity and covariates such as age, BMI, and gender. Principal coordinates analysis (PCoA) was performed based on the microbial species or pathway relative abundance by using the R program “ade4” package. We used the R program “vegan” package to calculate the Shannon index, the Simpson index, and the observed species number (OBS) in each sample and performed a permutational multivariate analysis of variance (PERMANOVA) with “adonis2.” The R program “qvalue” was used to perform multiple tests.

## Results

### Characteristics of the Primary Cohort

**Figure 1:**
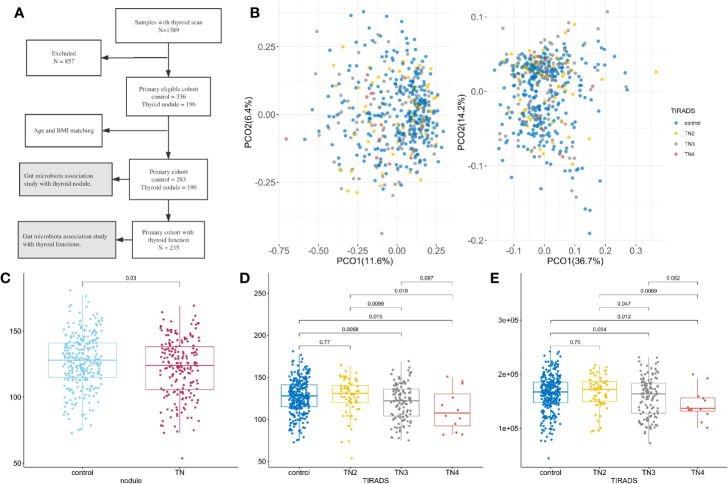
Microbiome composition and diversity. For all boxplots in this study, center is median, box is interquartile range (IQR), whisker is 1.5 × IQR. **(A)** Metagenomics Genome-Wide Association Study on the primary cohort; **(B)** Principal coordinate analysis with Spearman’s coefficient distance based on relative abundance of species (left panel) and pathways (right panel). Colored dots represent samples with TI-RADS grade, x-axis is PCO1, and y-axis is PCO2. Control (n = 283): no obvious nodules found in thyroid ultrasound. TN2 (n = 63): TI-RADS level 2. TN3 (n = 121): TI-RADS level 3. TN4 (n = 12): TI-RADS level 4a; **(C–E)** Boxplot showing distribution of microbial characteristics. Significance determined by single-sided Wilcoxon rank sum test. **(C)** Number of observed species (OBS) in control and TN; **(D)** OBS in TI-RADS; **(E)** Number of observed gene families in TI-RADS.

The primary cohort included 479 participants (40.71% women) after all exclusions (see **Figure 1A** and methods section). At least one thyroid nodule was detected in 196 (40.91%) participants. The mean ages of the control and TN groups were 43.42 and 43.79 years old, respectively. The participants were classified into four groups according to the thyroid ultrasonographic findings: (1) 283 demonstrated no remarkable findings (controls); (2) 63 had Thyroid Imaging Reporting and Data System (TI-RADS) level 2 (TN2); (3) 121 had TI-RADS level 3 (TN3); and (4) 12 had TI-RADS level 4a (TN4), of which five TI-RADS level 4a underwent fine-needle aspiration biopsy, which did not identify malignancy. Participants with more than one thyroid nodule were classified according to the highest TI-RADS level. The demographic and laboratory test result data are summarized in **Table S1**.

Compared with controls, females with TN had higher blood pressure and waist circumference (p < 0.05), and males with TN had higher triglyceride (TC), total cholesterol (TG), and high-density lipoprotein-cholesterol (HDL-C) concentrations. No significant differences were found between the TN2 and control groups, or between the TN3 and TN4 groups, except for higher blood pressure in female TN2 and TN4 groups (**Table S2**). Thyroid function and lifestyle habits, including smoking and drinking, were not related to TN incidence, except for the male TN group that had a lower FT3 concentration.

### Gut Microbial Characteristics of the Primary Cohort

A total of 309 microbial species, 415,928 gene families, and 371 metabolic pathways present in at least 30 fecal samples were used for downstream analysis. Of these, 31 species and 147 pathways had a median relative abundance >0.1%, comprising 44.63% of the species and 90.65% of the pathways in the total microbiome. The presence of thyroid nodules was significantly associated with microbiome pathway and species profile when using permutational multivariate analysis of variance (PERMANOVA, p < 0.05) with multiple distances including the Jensen-Shannon divergence, Bray–Curtis dissimilarity, Spearman’s coefficient, and Pearson’s correlation coefficient. As illustrated in **Figure 1B**, principal coordinates analysis (PCOA) conducted on the basis of species and metabolic pathway composition showed that the main principal coordinates (PCO1 or PCO2) correlated with the presence of thyroid nodules (Wilcoxon rank sum test, p < 0.05) and TI-RADS level (Kruskal-Wallis rank sum test, p < 0.05). The TN2 group was more similar to the controls than to the TN3 and TN4 groups with regard to inter-group distances (Wilcox rank sum test, p < 0.05, **Figure S2**). Moreover, no significant differences were found between the control and TN2 groups when comparing the distribution of the main PCOs (**Figure S1**).

### Lower Microbial Richness in Patients With Thyroid Nodules

After filtering out species and gene families with low frequency, the number of observed species (OBS) was significantly lower in the patients with thyroid nodules compared to the controls (Wilcoxon rank sum test, p < 0.05, **Figure 1C**). The numbers of OBS and observed gene families (OBG) were also lower in the TN2 group compared with the TN3 and TN4 groups (p < 0.05, **Figures 1D, E**), however, no significant differences in OBS or OBG were found between control and TN2 groups. We used generalized liner models (GLM) to confirm the reduction in microbial richness in the TN3 (p = 0.0025) and TN4 groups (p = 0.010) after adjusting for age (p = 0.10), sex (p = 0.16), and BMI (p = 0.014).

### TI-RADS Level 2 Group Was More Similar to Controls

**Figure 2:**
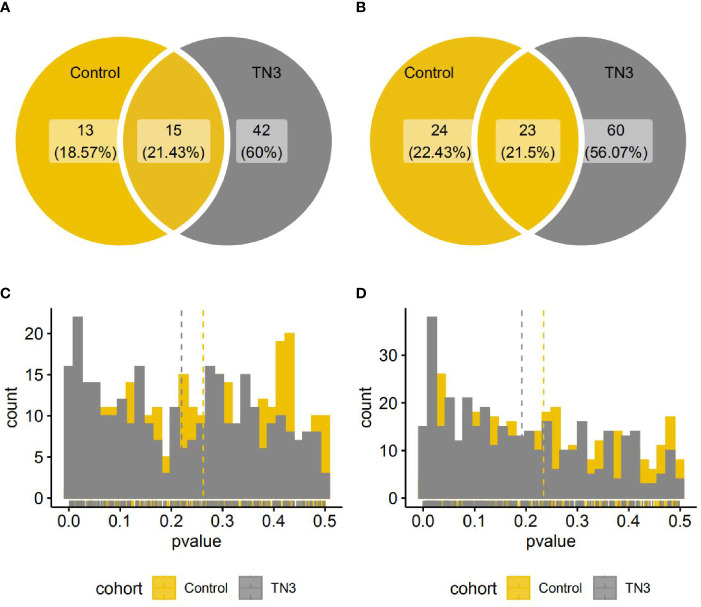
Species and pathways with significant different distribution between the TN2 and control or TN3 groups. **(A, B)** Venn diagrams indicate number of species **(A)** or pathways **(B)** with significantly different distributions. (One single-sided Wilcoxon rank sum test, p < 0.05); **(C, D)** Histograms indicate distributions of p value (One single-sided Wilcoxon rank sum test) in all species **(A)** or pathways **(B)**.

There was a significant difference between the TN2 and TN3 groups in the relative abundance of 57 species and 83 pathways (single-sided Wilcoxon rank sum test, p < 0.05), and in 28 species and 47 pathways between the TN2 and control groups. Of these, 15 species and 23 pathways commonly presented significant different distribution (**Figure 2**). As previously reported, we found that the TN2 group was more similar to the control group than to the TN3 and TN4 groups with respect to overall microbial distances. Therefore, we classified the control and TN2 groups as having no or low grade TIRADS (LTN) and those with TN3 or TN4 as having advanced or high grade TIRADS (HTN) for downstream analysis.

### Gut Microbiome Changes in Patients With High-Grade Thyroid Nodules

**Figure 3:**
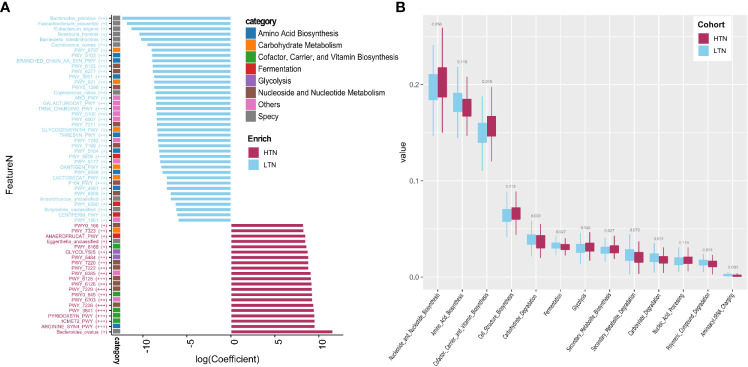
Microbiome associated with thyroid nodule group. Significance determined by Maaslin2 q value. **(A)** Statistically significant associated species and pathways. X-axis is transformed coefficient generated using Maaslin2, color bars attached to y-axis indicate pathway categories and species, significance is denoted in brackets as follows: +++, q value < 0.01; ++, q value 0.01–0.05; +, q value 0.05–0.15. Coefficient transformation was performed as: abs(co) × 1e7 × b, abs(co) is absolute value of coefficient, b is 1 or −1 when coefficient is positive or negative; **(B)** Boxplots showing distribution of pathway function categories in LTN (n = 346) and HTN (n = 133).

We used a combination of the Wilcoxon rank sum test and microbiome multivariable association with linear models (MaAsLin) to find microbial features associated with the presence of thyroid nodules. By adjusting for covariates (gender, age, and BMI) and controlling for multiple testing, 11 species and 52 pathways were found to be significantly associated with the presence of high-grade thyroid nodules (two-sided Wilcoxon rank sum multiple testing q value <0.05, MaAsLin adjusted q value <0·15) (**Figure 3A**, **Tables S3** and **S4**), and these comprised 14.25 and 23.99% of the total microbial composition, respectively. *Butyrivibrio unclassified*, *Bacteroides plebeius*, *Coprococcus comes*, *Coprococcus catus*, *Roseburia hominis*, *Eubacterium eligens*, *Anaerotruncus unclassified*, *Faecalibacterium prausnitzii*, and *Barnesiella intestinihominis* were enriched in the LTN group. However, the relative abundances of *Bacteroides ovatus* and *Eggerthella unclassified* were absent in the LTN group. Total amino acid biosynthesis, carbohydrate degradation and fermentation related pathways were also overrepresented in the LTN group. Metabolic pathways that were enriched in the HTN group included cofactor or vitamin biosynthesis, cell structure biosynthesis, nucleoside and nucleotide biosynthesis, and glycolysis (**Figure 3B** and **Table S5**).

### Reduced Abundances of Butyrate Production Pathways in Patients With Low-Grade Thyroid Nodules

**Figure 4:**
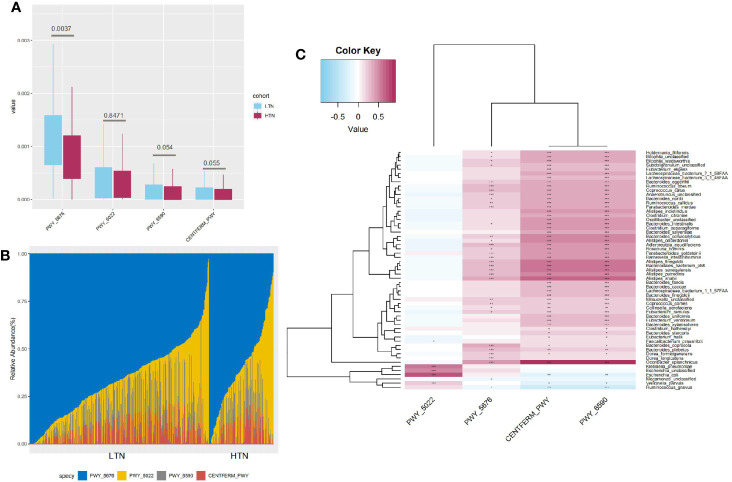
Characteristics of butyrate production pathways. **(A)** Boxplot showing distribution of butyrate production pathways, q value significance determined in Maaslin2; **(B)** Bar graph indicates composition of four different butyrate production pathways; **(C)** Heatmap indicates statistically significant correlations between butyrate production pathways and microbial species. Gradient color in heatmap indicates Spearman’s correlation coefficient, significance is denoted in cells as follows: +++, p value < 1e-5; ++, p value < 1e-4; +, p value < 1e-3, p value determined by using Spearman’s correlation test.

Four butyrate formation pathways were detected in our participants. An additional two pathways, P162-PWY (L-glutamate degradation) and P163-PWY (L-lysine fermentation), were also found, but as they had a median relative abundance lower than 1e-5, they were not further included in downstream analyses. PWY-6590 (pyruvate fermentation), PWY-5676 (acetyl-CoA fermentation), and CENTFERM-PWY (pyruvate fermentation) were enriched in participants with LTN. PWY-5022 (butanoyl-CoA fermentation) showed no difference in its distribution (**Figure 4A**). PWY-5676 was the main butanoate producing pathway, comprising 64.96% (range 2.81–100·00%, standard deviation 20.13%) of total butyrate productivity (**Figure 4B**). The butyrate producers enriched in the LTN group included *Coprococcus comes*, *Coprococcus catus*, *Roseburia hominis*, and *Eubacterium eligens*, which were also positively correlated with the butyrate producing pathways (**Figure 4C** and **Table S6**) (p < 0.001, Spearman’s correlation test).

### Association Between Gut Microbiome and Thyroid Function

In total, 235 participants with thyroid function test results were used to evaluate the association between gut microbiome and thyroid function. Using multivariate association with linear models (MaAsLin) and after adjusting for covariates (age, BMI, and gender), 12 pathways were associated with TSH (MaAsLin q value < 0·15) (**Table S7**).

L-histidine biosynthesis (HISTSYN-PWY) was positively associated with serum TSH. L-histidine is an essential component of thyrotropin releasing hormone (TRH) and could, therefore, increase TSH synthesis and secretion from the anterior pituitary. Conversely, L-histidine degradation (PWY-5030) was negatively correlated with TSH. In addition, biotin (vitamin H) biosynthesis was positively associated with FT3 (Spearman’s correlation test, p < 0.05) but did not withstand age adjusting. Interestingly, biotin, which is commonly used, might induce abnormal thyroid function test results (Ardabilygazir et al., 2018) *via* its direct effect on increasing thyroid hormone and reducing TSH concentrations (Katzman et al., 2018; Odhaib et al., 2019).

## Discussion

The presence of thyroid nodules (TN) is the most common watchful waiting endocrine disorder in clinical practice. Although patients with TN are not considered to be sick, TN has a complex and unknown etiology. Here, we described the gut microbial characteristics of patients with TN and the association between the presence of TN and thyroid function.

We found that the presence of TN was associated with lower gut microbial species and gene richness. Overall, microbial composition was associated with the presence of thyroid nodules and the TI-RADS level. By contrast, Zhang and colleagues (Zhang et al., 2019) reported increased species richness in TN and dramatic alterations in the overall microbial structure based on 16s rRNA gene sequencing and operational taxonomic units (OTU) profiling. In their study, TI-RADS or other thyroid nodule assessments were not available, making it difficult to compare these differing findings based on different sequencing methods.

We found that the typical butyrate-producing gut microbiota, including *Butyrivibrio unclassified*, *Coprococcus comes*, *Coprococcus catus*, *Roseburia hominis*, *Eubacterium eligens*, and *Faecalibacterium prausnitzii*, were reduced in patients with HTN. Previous studies reported that butyrate producers such as *Butyricimonas* were not present in individuals with thyroid nodules (Zhang et al., 2019) and that there was decreased short chain fatty acid (SCFA) producing ability in primary hypothyroidism (Steliou et al., 2012). Zhang and colleagues (He et al., 2019) reported that metformin treatment could induce thyroid nodule size reduction, which could also promote a fecal SFC-producing gut microbiome (Wu et al., 2017).

In this study, we found higher butyrate production and amino acid biosynthesis in patients with LTN. The microbiome in the human colon can produce butyrate in several ways (Louis and Flint, 2017), with the majority of butyrate formed *via* acetyl-CoA fermentation (PWY-5676), which is the main butyrate formation pathway in the human gut microbiome (Vital et al., 2014). Amino acid biosynthesis can promote butyrate formation *via* the intestinal microbiome (Neis et al., 2015; Rios-Covian et al., 2017), and several studies have shown that butyrate production increases with the addition of the amino acids isoleucine (PWY-3001) (Zhang et al., 2013) and threonine (THRESYN-PWY) (Gaifem et al., 2018). The superpathway of branched chain amino acid (BRANCHED-CHAIN-AA-SYN-PWY) and chorismate biosynthesis (ARO-PWY), essential to aromatic amino acid biosynthesis, was also enriched in patients with LTN, as was the transfer RNA charging pathway (TRNA-CHARGING-PWY) which incorporates amino acids into growing polypeptides. Several metabolomic studies have shown the accumulation of L-glutamate (Huang et al., 2019) and lactate (Ryoo et al., 2016; Gomes and Gebrim, 2018) in benign thyroid nodules, which might be due to an increase in glycolysis (PWY-5484) and homolactic fermentation (ANAEROFRUCAT-PWY) related pathways.

As a well-recognized histone deacetylase inhibitor, butyrate can induce growth arrest in a variety of normal cell types and increase iodine uptake (Provenzano et al., 2007) in thyroid follicular carcinoma cells by restoring the human sodium-iodide symporter function (NIS). Butyrate has also been shown to increase thyroid hormone receptor expression in mice GH3 (Lazar, 1990) and mice glial C6 (Ortiz-Caro et al., 1986) cells. Benign nodules are characterized by reduced expression of NIS mRNA (Yim et al., 2019) and an intermediate epigenetic pattern between normal thyroid tissues and malignant nodules (Sodreé et al., 2008); normal epigenetic expression could be restored by sodium butyrate-induced demethylation (Shin et al., 2012).

In conclusion, the present study described the characteristics of the gut microbiome in patients with thyroid nodules and the relationship between the gut microbiome and thyroid functions. We found that alterations in gut microbiome functions in patients with high-grade thyroid nodules. Notably, we found that key gut microbiome-driven metabolic pathways, such as butyrate formation, were associated with the presence of thyroid nodules. Although these associations need further evaluation, we have demonstrated that the gut-thyroid link is likely mediated *via* microbial nutrition metabolism, which suggests that specific gut microbiota may be potential therapeutic targets to regulate thyroid metabolism.

## Data Availability Statement

The datasets presented in this study can be found in online repositories. The names of the repository/repositories and accession number(s) can be found below: https://www.ebi.ac.uk/ena, PRJEB36271.

## Ethics Statement

The studies involving human participants were reviewed and approved by the ethics committee from the First Affiliated Hospital of Zhengzhou University. The patients/participants provided their written informed consent to participate in this study.

## Author Contributions

Conceptualization, SD. Methodology and formal analysis, AL and JC. Resources, TL, XG, HY, MH, LW, DY, HL, and RM. Original draft preparation, AL and JC. Writing, review, and editing, SD. Visualization, AL. Project administration, QZ and SD. All authors contributed to the article and approved the submitted version. AL, JC, TL, XG, and HY contributed equally to this study.

## Funding

This study was equally funded by Chinese National Science and Technology Major Project 2018ZX10305410, Henan Province Key Scientific Research Projects of Universities 21A320035.

## Conflict of Interest

The authors declare that the research was conducted in the absence of any commercial or financial relationships that could be construed as a potential conflict of interest.

## Supplementary Material

Supplementary Table 1 Demographic characteristics in control and thyroid nodule groups.

Supplementary Table 2 Demographic characteristics in control and TN2 groups, or TN3 and TN4 groups.

Supplementary Table 3 Microbial species associated with thyroid nodule group.

Supplementary Table 4 Microbial pathways associated with thyroid nodule group.

Supplementary Table 5 Microbial function categories associated with thyroid nodule group.

Supplementary Table 6 Species associated with butyric acid production pathway.

Supplementary Table 7 Microbial pathways associated with thyroid function tests.
