# Supplementary material for: Gut Microbiome Alterations in Patients With Thyroid Nodules
Source: Front Cell Infect Microbiol. 2021 Mar 12;11:643968. doi: 10.3389/fcimb.2021.643968 (PMC8005713; doi:10.3389/fcimb.2021.643968)
Supplement: Supplementary file 2 [file DataSheet_1.docx]

Supplementary information


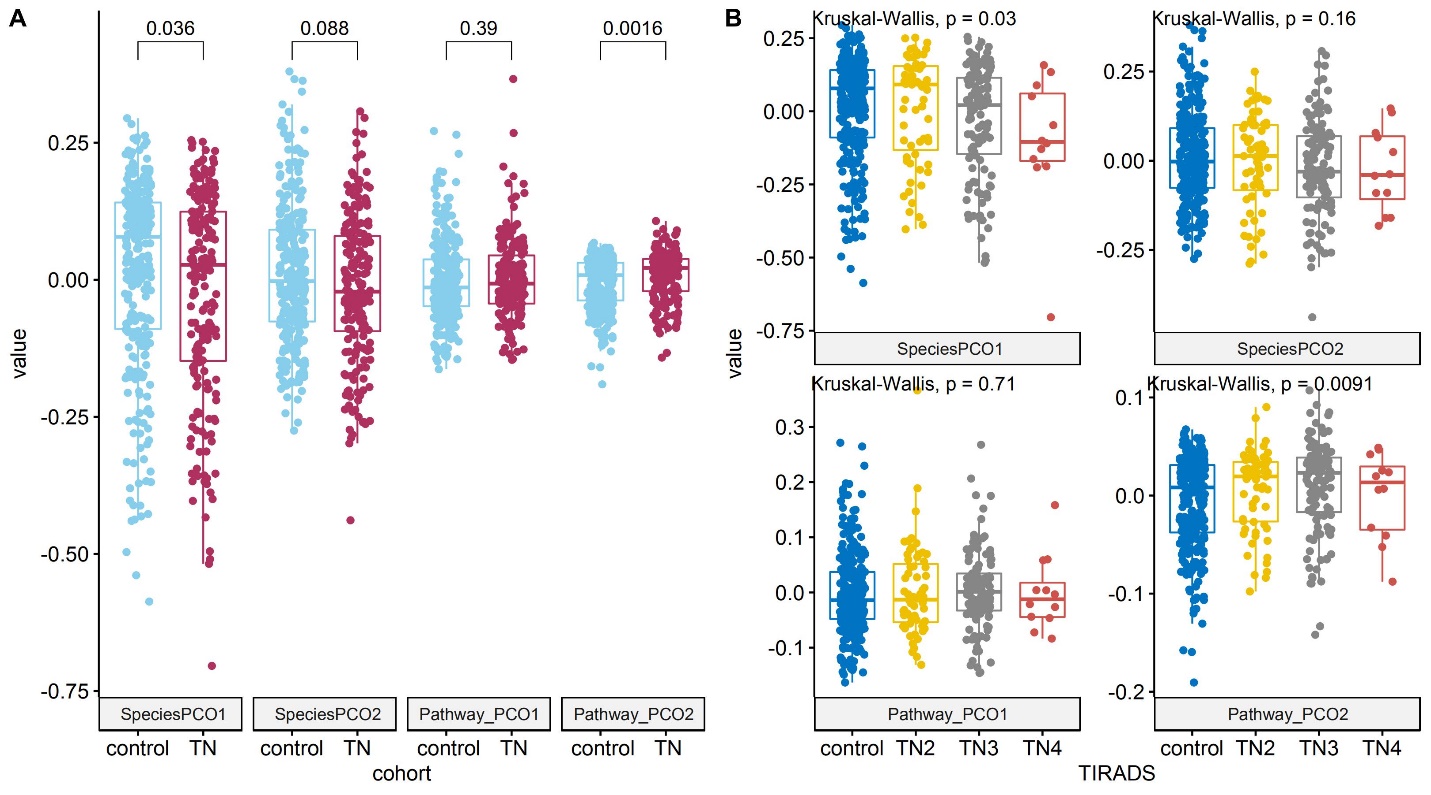


**Figure S1.** **Distribution of main principal coordinates (PCOs) in all samples**. Specy PCO1 and specy POC2 denote the main PCOs generated with microbial specy composition. Pathway PCO1 and pathway PCO2 denote the main PCOs generated with pathway composition. (a) Significance determined by two sided Wilcoxon rank sum test bewteen control and thyroid nodule (TN); (b) Significant determined by Kruskal-Wallis rank sum test among control and TI-RADS levels.


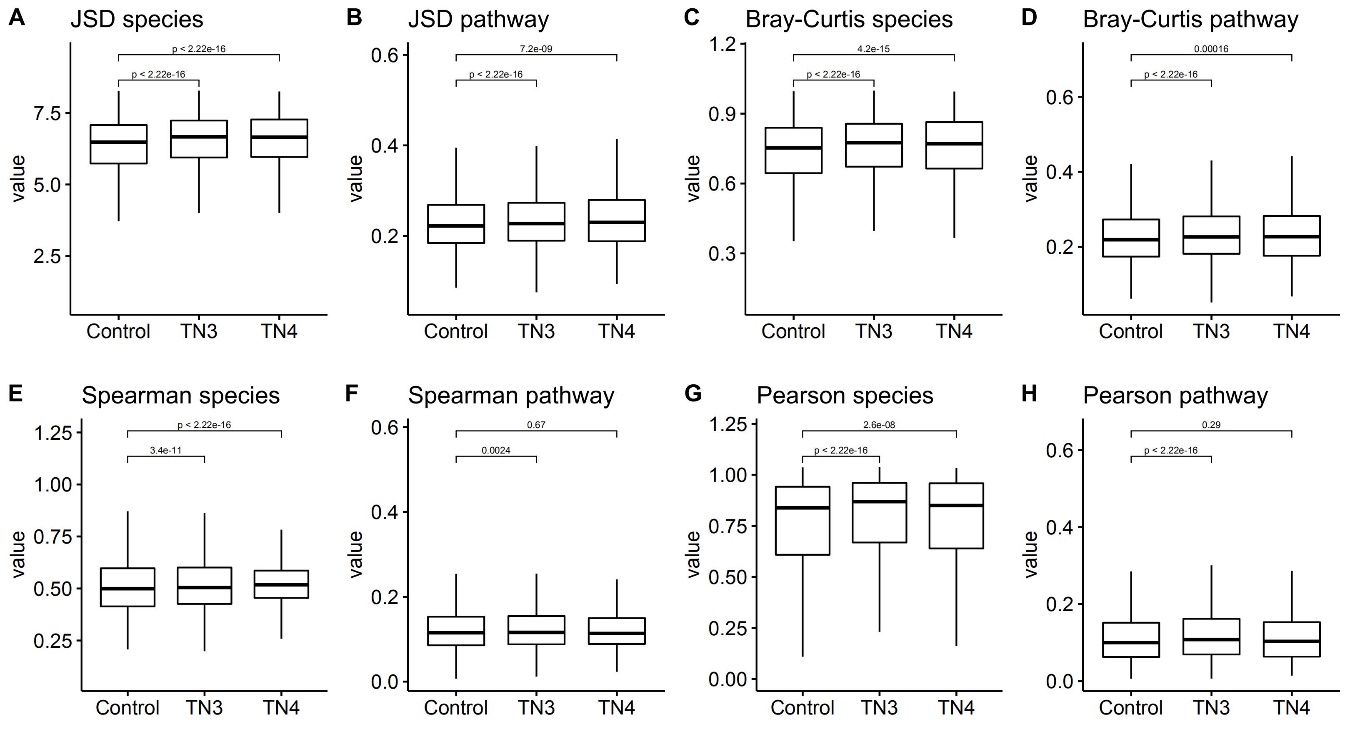


**Figure S2. Distribution of distances between TN2 with Control, TN3 and TN4.** Each panel indicated distances between TN2 and other groups including Control, TN3 and TN4, x-axis were groups, y-axis was distance value of combination of distance type and microbial composition (specy or pathway), significance determined by single sided Student’s t test. (a) JSD distance of species; (b) JSD distance of pathways; (c) Bray distance of species; (d) Bray distance of pathways; (e) Spearman coefficient distance of species; (f) Spearman coefficient distance of pathways; (g) Pearson Coefficient Distance of species; (h) Pearson Coefficient Distance of pathways. Significance determined in
